# Comprehensive Risk Assessment of Applying Biogas Slurry in Peanut Cultivation

**DOI:** 10.3389/fnut.2021.702096

**Published:** 2021-10-14

**Authors:** Qingyu Liu, Zixuan Zhao, Zhiping Xue, Ding Li, Zhining Wen, Yi Ran, Zili Mei, Li He

**Affiliations:** ^1^College of Engineering, Shenyang Agricultural University, Shenyang, China; ^2^Key Laboratory of Development and Application of Rural Renewable Energy, Biogas Institute of Ministry of Agriculture and Rural Affairs, Chengdu, China; ^3^Institute of Development Studies, Southwestern University of Finance and Economics, Chengdu, China; ^4^College of Chemistry, Sichuan University, Chengdu, China

**Keywords:** risk assessment, biogas slurry, heavy metal, peanuts, cultivation

## Abstract

Biogas slurry, a byproduct of biogas plants, is considered a high-quality bio-organic fertilizer. Despite providing nutrients to crops, biogas slurry may contain a high concentration of heavy metals, leading to food safety problems and endangering human health if such metals are absorbed by plants. Therefore, biogas slurry should undergo systematic risk assessment prior to direct use on farmland to ensure its safety for soils and crops. In this study, the risk of applying biogas slurry in peanut cultivation was comprehensively evaluated. Based on nitrogen contents, different concentrations of biogas slurry were applied in peanut cultivation. The results achieved herein showed that the application of biogas slurry as a nutrient supplier in peanut cultivation would significantly affect the physical and chemical properties of soil and characteristics of the plant and the quality of peanuts. Although the heavy metal content of biogas slurry was within the permitted range, it had potential risks to human health and the environment. Principal component analysis (PCA) showed that biogas slurry was the primary source of heavy metals in soil. After the application of biogas slurry, the contents of As and Hg in the soil increased significantly, which were 11.12 and 26.67 times higher than those in the control soil. The contents of Cu, Zn, Pb, Cd, and As in peanut kernel samples under different levels of biogas slurry application were all lower than the maximum permissible limit set by the Standardization Administration of China. In contrast, the content of Hg in peanut kernels was higher than the maximum permissible limit value of 0.02 mg/kg. Peanut had a higher enrichment capacity of Cd and Zn and a higher migration capacity of Pb. The health risk assessment showed that the long-term consumption of peanuts grown with a high dosage of biogas slurry would be harmful to the health of children aged 2–6 years with a large consumption level.

## Introduction

The dry matter and organic dry matter produced daily by livestock worldwide can exceed 20 and 10 million tons, respectively (1); thus, livestock and poultry wastes are the main sources of agricultural non-point-source pollution around the world (2). With a sharp increase in intensification of livestock and poultry farming, China has set strict regulations on both size and location of pig farms to reduce the associated environmental pollution and achieve global sustainable development. At present, the treatment of livestock and poultry manure includes mainly physical methods [sedimentation (3), filtration (4), dry and wet separation (5), and high-temperature drying (6)], chemical methods [precipitation (7), flocculation (8), and advanced oxidation processes (9)], and biochemical methods [aerobic (10) and anaerobic fermentation (11)]. Among these methods, anaerobic fermentation has been receiving large attention for being simple, inexpensive, and environment friendly (12).

The anaerobic fermentation process leads to the formation of a huge amount of biogas slurry that is rich in inorganic elements, such as nitrogen, phosphorus, potassium, and trace elements. The organic components of biogas slurry, such as amino acids, vitamins, and beneficial bacteria, make it suitable as a soil amendment (13). However, a large number of studies have shown that the long-term application of untreated biogas slurry to farmland would bring about crop failure, with weak growth and poor quality. Moreover, the accumulation of heavy metals and antibiotic resistance genes in soil and crops may be transferred to the human body via the food chain, affecting human health and potentially damaging organ functions (14). The overdose of trace elements (such as copper, zinc, and lead), veterinary antibiotics, and other feed additives has contributed to such phenomena (15). Therefore, it is of high importance to assess the risks of biogas slurry application and control the range of application rates (16).

Peanut (*Arachis hypogaea* L.) is one of the most important oil crops in the world, grown in more than 100 countries, especially in developing countries; it ranks as the second oil crop next to rapeseed (17). Currently, the global peanut cultivation area is estimated at 22.67 million ha with an annual production of 35 million tons (18). As it is rich in proteins, lipids, carbohydrates, minerals, phospholipids, vitamins, and other bioactive ingredients, peanuts play an important role in daily life (19). They are recognized as one of the healthy foods for their comprehensive and relatively balanced nutritional components (20).

As an underground fruiting crop, the root system of peanuts has a strong absorption capacity, resulting in a higher concentration of heavy metals in both shoots and fruits compared with other crops (21). In addition, the kernels can also absorb large amounts of trace elements. Due to soil pollution in planting areas (e.g., due to sewage irrigation, improper fertilization, and excessive pesticides application), the occurrence of excessive contents of heavy metals in peanut production areas is frequent (22). Although some scholars have already investigated the heavy metal pollution in the soils of peanut planting regions (18, 21, 23), no study could be found on the safety risk assessment of applying biogas slurry on the soil and peanut quality.

In light of the abovementioned reasons, the peanut was selected as the field test crop in this study. The potential ecological risks of heavy metals in soil and the growing risks to peanuts under different nitrogen application rates were evaluated. In addition, the estimated daily Intake (EDI) of peanuts and the health risks associated with selected contaminants ingested through peanuts were calculated. The results of this study would help provide a reference for the conversion of biogas by-products into organic fertilizers, which is crucial for the healthy expansion of the peanut industry and the development and implementation of more effective agricultural strategies.

## Materials and Methods

### Materials and Area

The experiment was conducted from July to November in 2019 at the Scientific Research Base of Shenyang Agricultural University (Shenyang City in Liaoning Province) and lasted for 100 days. Shenyang is located in the southern part of northeast China, with the humid to sub-humid, warm temperate, continental monsoon climate, with plain landform. Peanut (varieties: Nonghua 11) was cultivated in loess brown soil. The biogas slurry used in this study had been collected from a swine manure biogas plant and stored for 2 months before the field application. No pesticide was applied during the experiment.

### Experiment Design and Sample Collection

#### Experiment Design

Each experimental plot was 42 m^2^ (6.50 m × 6.50 m) and had five equal cropping areas. Two planting ridges (6.20 m × 0.60 m) were set up in each zone, and 10 crops were planted in each row. The row spacing was >0.45 m, and the plant spacing was >0.30 m. An isolation zone was set up between the test plots to avoid the mass transfer between plots. Reasonable application of nitrogen fertilizer is of great significance for the growth of peanuts because the amount of nitrogen required will vary greatly with the different planting methods, planting conditions, and variety yield, the problem of nitrogen fertilizer amount for peanuts has been a concern to scholar for a long time (24, 25). According to different nitrogen rates of biogas slurry, the experimental plots were divided into five groups, including no fertilization, low application rate [8.5 g total nitrogen (TN)/m^2^·year], European Union application standard (17 g TN/m^2^·year), domestic average application rate (34 g TN/m^2^·year) (26), and excessive application (68 g TN/m^2^·year).

#### Samples Collection

Before and after planting, the S-shaped sampling method was used to collect soil samples in the 0–30 cm layer, through which the soil background value was recorded as S_orig_. After the complete harvesting of the peanuts, soil samples from different plots were recorded as S_0_ (soil sample from no fertilizer land), S_8.5_ (soil sample from 8.5 g TN/m^2^·year land), S_17_ (soil sample from 17 g TN/m^2^·year land), S_34_ (soil sample from 34 g TN/m^2^·year land), and S_68_ (soil sample from 68 g TN/m^2^·year land). After removing weeds, soil pests, stones, and other impurities, all soil samples were naturally air dried, then crushed and passed through a 100-mesh sieve. Table 1 gives the basic physical and chemical properties of the biogas slurry and soil before and after planting.

**Table 1:** Basic physical and chemical properties of the tested soil and biogas slurry.

	**Biogas slurry**	**S_**orig**_**	**S_**0**_**	**S_**8.5**_**	**S_**17**_**	**S_**34**_**	**S_**68**_**
pH	7.72 ± 0.01	5.76 ± 0.13^b^	5.81 ± 0.09^b^	6.21 ± 0.02^a^	6.24 ± 0.09^a^	6.24 ± 0.22^a^	6.35 ± 0.01^a^
OM (g/kg)	172.8 ± 3.32	13.40 ± 3.10^a^	12.84 ± 1.25^a^	12.30 ± 0.83^a^	10.89 ± 0.95^ab^	8.20 ± 2.33^b^	11.47 ± 0.82^ab^
TN (g/kg)	5.25 ± 0.58	2.05 ± 0.21^a^	1.55 ± 0.11^b^	1.43 ± 0.09^b^	1.57 ± 0.26^b^	1.62 ± 0.11^b^	1.46 ± 0.16^b^
TP (g/kg)	4.98 ± 0.18	0.65 ± 0.01^ab^	0.68 ± 0.00^a^	0.62 ± 0.04^bc^	0.60 ± 0.07^c^	0.63 ± 0.02^bc^	0.62 ± 0.02^bc^
TK (g/kg)	4.95 ± 1.24	17.73 ± 0.10^a^	17.69 ± 0.16^a^	17.82 ± 0.10^a^	18.18 ± 0.72^a^	18.13 ± 0.31^a^	18.24 ± 0.05^a^
Avail.N. (mg/kg)	670.12 ± 60.64	225.27 ± 29.64^a^	137.63 ± 19.60^b^	113.28 ± 5.08^b^	107.92 ± 12.09^b^	123.82 ± 14.88^b^	128.90 ± 17.40^b^
Avail.P. (mg/kg)	91.61 ± 1.06	15.39 ± 0.29^b^	14.98 ± 0.43^c^	14.85 ± 0.10^c^	13.42 ± 0.20^d^	16.72 ± 0.16^a^	14.71 ± 0.05^c^
Avail.K. (mg/kg)	3346.84 ± 238.77	237.40 ± 0.00^a^	219.08 ± 4.07^bc^	207.68 ± 2.65^d^	193.14 ± 2.50e	220.62 ± 1.66^b^	213.59 ± 2.46^cd^

*Lowercase letters followed by numbers in the same line indicate significant differences at the level of 0.05. Marked with different letters means the difference is significant, marked with the same letter means the difference is not significant.

At maturity, all the plants were harvested, and the samples of roots, stems, leaves, shells, and kernels of the peanut plants were collected separately and kept at −80°C for 12 h followed by freeze-drying and storage at −80°C for later use.

### Chemical Analyses

The alkali-hydrolyzed nitrogen (Avail.N.), available phosphorus (Avail.P.), and available potassium (Avail.K.) in biogas slurry were determined according to the Agricultural Standard of the People's Republic of China (27–29). pH (30) was measured by a Seven Compact pH meter (Mettler Toledo, Switzerland). Organic matter (OM) was measured by the potassium dichromate method (31). TN (32) in soils and protein (33) in peanut kernels was determined by an automatic Kjeldahl nitrogen determination apparatus (KD-310, Opsis, Sweden), whereas the hydrolysis diffusion method was used to determine Avail.N. Total phosphorus (TP) and Avail.P. were determined by a spectrophotometer (UV-2600, Shimadzu, Japan). The soil, biogas slurry, and peanut samples were digested by acids (HNO_3_-HClO_4_-HF). The contents of Cu, Zn, Pb, Cr, total potassium (TK), Avail.K., and Cd in the samples were determined by atomic absorption spectrophotometry (PinAAcle 900T, PerkinElmer, Waltham, MA, USA). The contents of As and Hg were determined by an atomic fluorescence spectrophotometer (AFS-922, Titan Instruments, Beijing). The main stem height and lateral branch length of peanut plants were directly measured, and the branch number and fruit number per plant were directly counted.

### Assessment of Potential Ecological Risk of Heavy Metals

#### Integrated Pollution Index (IPI_N_)

The pollution index for single heavy metals (*PIi*) and *IPI*_*N*_ can be used to quantitatively evaluate the extent of heavy metal pollution of soil (34). These indices are calculated using (Equations 1, 2) as follows:


(1) $$\begin{array}{l} PI_{i}=\frac{C_{i}}{T_{i}} \end{array}$$

(2) $$\begin{array}{l} IPI_{N}={\left[\frac{\left({IPI_{avg}}^{2}+{IPI_{\max}}^{2}\right)}{2}\right]}^{\frac{1}{2}} \end{array}$$

where *C*_*i*_ is the measured content of element *i* in soil, *T*_*i*_ is the management target value of soil element *i*, i.e., the reference value in the “Soil Environmental Quality Risk Control Standard for Soil Contamination of Agricultural Land” (Supplementary Table 1) and the parameters are as follows: Zn = 200, Cr = 150, Cu = 50, As = 40, Cd = 0.3, Pb = 90, and Hg = 1.8 (35). *IPI*_*avg*_ is the average value of *PIi* of all heavy metal elements involved, and *IPI*_max_ is the maximum value.

#### Potential Ecological Risk Index **(*RI*)**

The potential ecological index method comprehensively considers the ecological and toxicological effects of heavy metals to evaluate the degree of soil heavy metal pollution and ecological risk (36), and its calculation formula is as follows:


(3) $$\begin{array}{l} RI=\overset{n}{\underset{i=1}{\sum }}E_{r}^{i}=\overset{n}{\underset{i=1}{\sum }}\left(T_{r}^{i}\times C_{f}^{i}\right)=\overset{n}{\underset{i=1}{\sum }}\left(T_{r}^{i}\times \frac{c_{i}}{c_{b}^{i}}\right) \end{array}$$

where $C_{f}^{i}$ is the pollution index of heavy metal, *C*_*i*_ is the measured value of heavy metal, and $C_{b}^{i}$ is the reference ratio of a given heavy metal; the heavy metal background value of the test field soil is used as the reference ratio. $E_{r}^{i}$ is the potential ecological risk index of heavy metals and $T_{r}^{i}$ is the toxicity response parameters of a certain heavy metal. According to the Hakanson potential ecological hazard index, the parameters are: Zn = 1, Cr = 2, Cu = Pb = 5, As = 10, Cd = 30, and Hg = 40 (37).

#### Biological Enrichment Factor **(*BCF*)**

Biological enrichment factor refers to the quantitative relationship incorporating the accumulation and absorption capacity of an organism (38). It is the ratio of the content of a certain element in an organism to the content of this element in its living environment and reflects the risk of, and a degree of damage from, heavy metal accumulation in crops. This experiment mainly explored the biological enrichment of heavy metals in peanut kernels using the following equation:


(4) $$\begin{array}{l} BCF=\frac{C_{i\ker nels}}{C_{isoil}} \end{array}$$

where *C*_*i*ker*nels*_ is the content of heavy metal *i* in the edible part of crops, *C*_*isoil*_ is the content of heavy metal *i* in soil.

#### Transfer Factor **(*TF*)**

Transfer factor is expressed as the ratio of the content of heavy metals in leaves to that in the roots of plants (39), which is a measure of the transfer of heavy metals between different plant parts. This experiment mainly explored the transport of heavy metals from roots to leaves and was calculated as follows:


(5) $$\begin{array}{l} TF=\frac{C_{ileaf}}{C_{iroot}} \end{array}$$

where *C*_*ileaf*_ is the content of heavy metal *i* in leaves, and *C*_*iroot*_ is the content of heavy metal *i* in roots.

### Health Risk Assessment

**EDI**

Estimated daily intake depends on the heavy metal concentration and daily food consumption of peanuts (40), and *EDI* (μg kg^−1^ d^−1^) is calculated as follows:


(6) $$\begin{array}{l} EDI=\frac{IR\times C}{W_{AB}}\times 10^{3} \end{array}$$

where *IR* is the intake rate of peanuts (kg/person per day; Supplementary Table 2), *C* is the concentration of heavy metals in peanuts (mg/kg), and *W*_*AB*_ is the average weight of a person (kg).

#### Total Hazard Quotient **(*THQ*)**

According to the U.S. Environmental Protection Agency (USEPA) standard methodology, the potential exposure risk of non-carcinogenic substances was indicated by the ratio of *EDI* to oral reference dose (41). This ratio is called *THQ*. *ADI* (the oral reference dose in mg kg^−1^ d^−1^) for various elements is: Cu 4 × 10^−2^, Zn 3 × 10^−1^, Pb 3.5 × 10^−3^, Cr 1.5, Cd 3 × 10^−3^, As 3 × 10^−4^, and Hg 5 × 10^−4^ (42, 43). *THQ* is calculated as follows:


(7) $$\begin{array}{l} THQ=\frac{EDI}{ADI} \end{array}$$

If the *THQ* value is <1, exposed individuals are unlikely to experience significant toxic effects. If it exceeds 1, the significant toxic effects are likely; as the *THQ* value increases, so does the likelihood.

#### Total Target Hazard Quotient (TTHQ)

Total target hazard quotient was used to evaluate the cumulative potential health risks associated with heavy metal accumulated in peanuts. *TTHQ* is calculated as follows:


(8) $$\begin{array}{l} TTHQ=\overset{i}{\underset{n=1}{\sum }}THQ_{n} \end{array}$$

If the *TTHQ* value is <1, the exposed person is unlikely to experience significant toxic effects; if at or above 1, there is a possibility of significant adverse effects due to heavy metal interactions, and this likelihood increases as the *TTHQ* value increases (44).

### Statistical Analysis

Microsoft Office 2019 was used for basic data processing. Origin 2021 was used for drawing figures and performing principal component analysis (PCA). SPSS 22 was used for significance analysis and Canoco5 was used for redundancy analysis.

## Results and Discussion

### Potential Ecological Risk Assessment of Biogas Slurry Application to Soil

#### Characteristics of Heavy Metal Content in the Soil in the Root Zone

**Figure 1:**
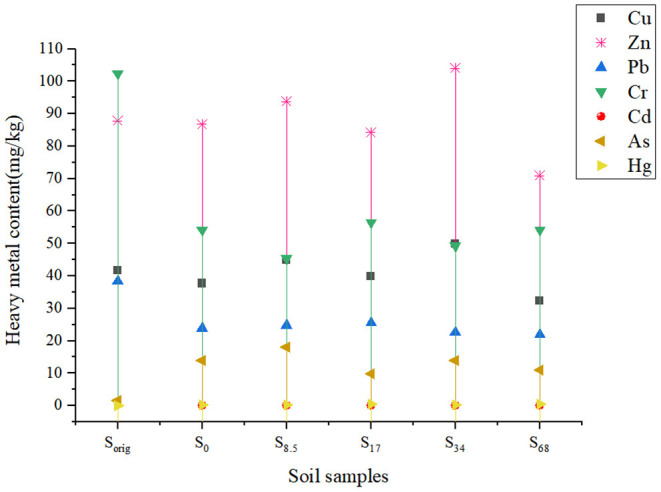
Heavy metal content in soil samples before and after planting.

The comparison between the content of heavy metals in the unplanted and planted soil is shown in Figure 1. Having considered the “Soil Environmental Quality Risk Control Standard for Soil Contamination of Agricultural Land” (35), it was concluded that the soil under consideration had a low risk because the content of heavy metals in each soil sample was smaller than the farmland soil pollution risk reference values. Based on the Chinese agricultural standard “Microbial Organic Fertilizers” (45), the content of heavy metals in biogas slurry used in this experiment did not exceed the permissible limits (Table 2). The contents of As and Hg in the cultivated soil treated with biogas slurry increased significantly, reaching the highest values of 18 and 0.48 mg/kg, respectively, which were 11.12 and 26.67 times higher than those in the control soil. Cd was not detected in the control soil, but its concentration increased in the soil treated with biogas slurry. Such an increase in the concentration of heavy metals in the soil due to the application of biogas slurry needs careful attention. The long-term application of large amounts of biogas slurry will likely cause serious soil heavy metal accumulation. Duan et al. found that after application of biogas slurry for 8 years, the concentration of As, Cd, Cu, and Zn was significantly higher than those in the control (46).

**Table 2:** The content of heavy metals in biogas slurry and heavy metals limitation for organic fertilizer.

**Test materials**	**Cu (mg/kg)**	**Zn (mg/kg)**	**Pb (mg/kg)**	**Cr (mg/kg)**	**Cd (mg/kg)**	**As (mg/kg)**	**Hg (μg/kg)**
Biogas slurry	97.86 ± 1.54	367.94 ± 2.68	4.45 ± 0.25	10.28 ± 0.14	0.05 ± 0.00	1.14 ± 0.15	8.49 ± 0.65
Limiting value (dry base)			≤ 50	≤ 150	≤ 3	≤ 15	≤ 2,000

Chen et al. showed that the fungal community composition of paddy soils treated with excessive rates of biogas digestate for a long time was affected by changes in the chemical properties and heavy metal contents (47). It has been shown that the application of biogas slurry could increase the abundance of microorganisms in the soil (48). Microorganisms convert heavy metals to a certain extent. For example, sulfate-reducing bacteria, abundant in biogas slurry, are the main mercury-methylation bacteria (49).

In contrast to As and Hg, the contents of Pb and Cr decreased significantly in different soil samples after the application of biogas slurry. This was attributed to plant absorption of these elements together with nutrients during growth. Plant absorption of some specific elements may be greater than the amount of these elements added via fertilization, leading to a decrease of Pb and Cr contents in the soil after cultivation (26).

#### Analysis of Soil Pollution Around Roots

The single factor pollution index *PI*_*i*_ has five grades in “Specification of Land Quality Geochemical Assessment” (50). The Nemero Composite Pollution Index *IPI*_*N*_ is divided into five levels in “The Technical Specification for Soil Environmental Monitoring” (51). In 1980, Hakanson proposed the potential ecological harm index RI with four levels. The evaluation results are shown in Supplementary Table 3.

**Figure 2:**
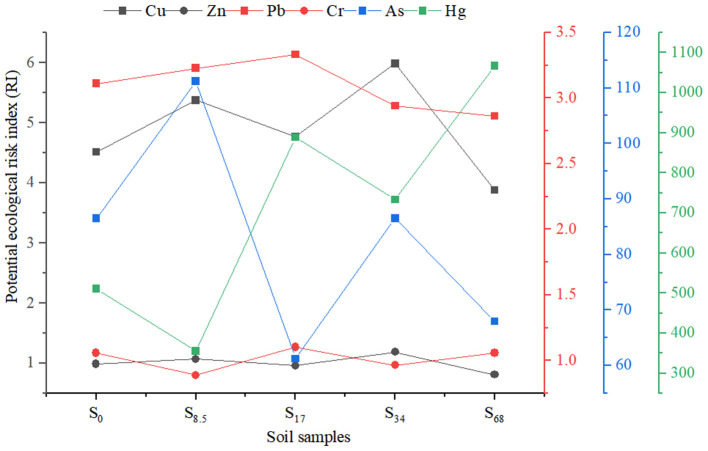
Index of potential ecological risk of heavy metals in soil samples after application of biogas slurry.

It can be seen from Table 3 that the integrated pollution coefficients of soil after applying biogas slurry are all <1, indicating that the soil is non-polluted after biogas slurry application (S_34_ is mildly clean). The potential ecological risk index of soil after applying biogas slurry was >300, indicating that the potential ecological risk to the soil after the application was high, which might have been caused by the high potential ecological risk index of Hg (Figure 2).

**Table 3:** Integrated pollution index (*IPI*_*N*_) and potential ecological risk index (*RI*).

**Indicator**	**S_**orig**_**	**S_**0**_**	**S_**8.5**_**	**S_**17**_**	**S_**34**_**	**S_**68**_**
*IPI*_*N*_	0.6568	0.5955	0.6935	0.6223	0.7656	0.5240
*RI*		607.3	477.4	960.2	830.9	1143.3

#### Analysis of Heavy Metal Sources in the Soil in the Root Zone

**Figure 3:**
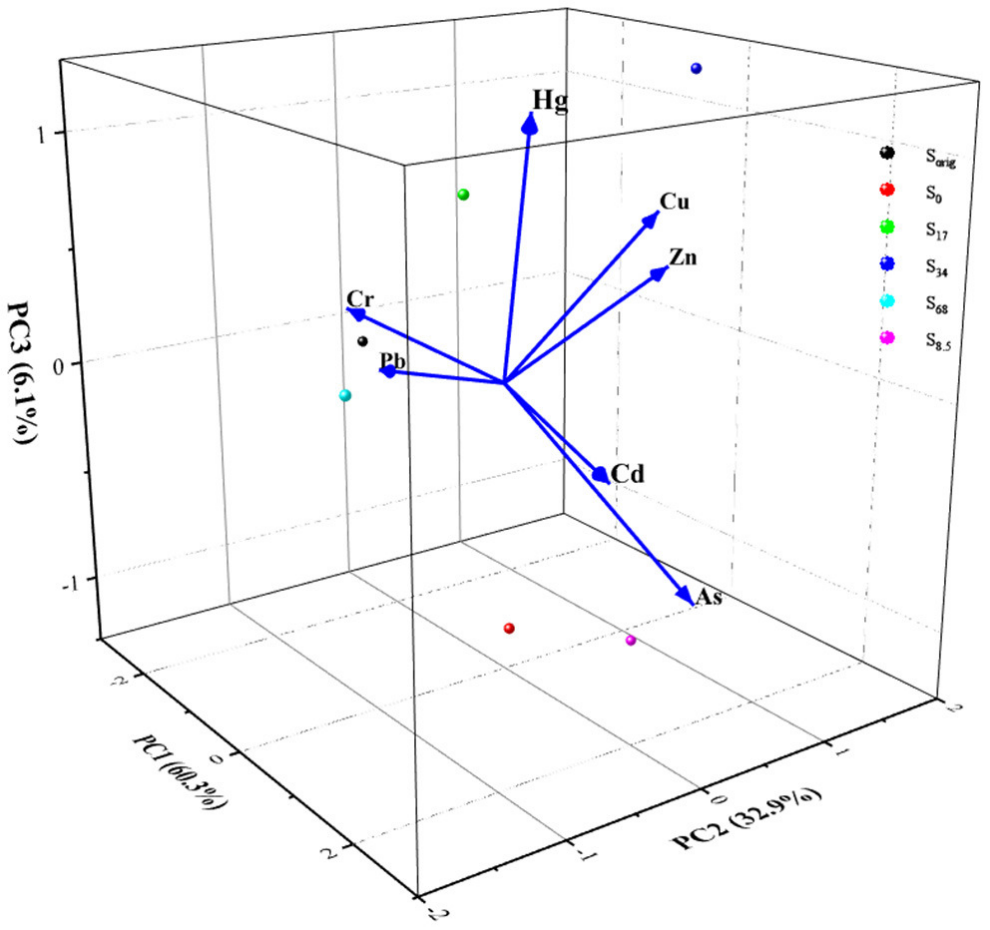
Principal component analysis of heavy metal content in rhizosphere soil.

Heavy metals in soil mainly come from the soil parent materials and human activities. PCA can effectively identify the pollution sources of heavy metals in soil (52). The PCA results of heavy metals in the experimental field soil in the present study are shown in Table 4. The first three principal components explained 99.260% of the variation in the heavy metal contents in the experimental field. Figure 3 shows the results of PCA of soil heavy metal content in the study area. The first principal component (PC1) explained 60.296% of the total variation, and the load on the content of Cd, Hg, and As was high (0.480, 0.403, and 0.382, respectively). Considering the results presented in section Characteristics of Heavy Metal Content in Soil in the Root Zone and those shown here, it is inferred that the three metals associated with the PC1 have the same source and are mainly affected by the application of biogas slurry.

**Table 4:** Matrix for principal component analysis loadings of soil heavy metals.

**Heavy metals**	**PC1**	**PC2**	**PC3**
Cu	−0.080	0.631	0.339
Zn	−0.054	0.645	0.196
Pb	−0.483	−0.047	−0.091
Cr	−0.468	−0.166	0.100
Cd	0.480	−0.083	−0.090
As	0.403	0.296	−0.505
Hg	0.382	−0.247	0.752
Eigenvalue	4.221	2.301	0.427
Percentage of variance (%)	60.296	32.870	6.093

The second principal component (PC2) explained 32.870% of the variation, with Cu and Zn having high loads, i.e., 0.631 and 0.642, respectively. Industrial dust, municipal sewage, chemical fertilizers, and pesticide use are the main contributors to Cu and Zn pollution of farmland soil, so it is speculated that the PC2 is mainly affected by human activities, such as sewage irrigation before the experiment.

The third principal component (PC3) explained 6.093% of the variation, and Cr had a high load. The results of the source analysis of Cr in different areas showed that Cr was less affected by human activities (53), so it was speculated that the PC3 was mainly affected by the natural geology of the test area.

### Peanut Growth Risk Assessment Due to Biogas Slurry Application

#### Distribution of Heavy Metals in Plant Organs

**Figure 4:**
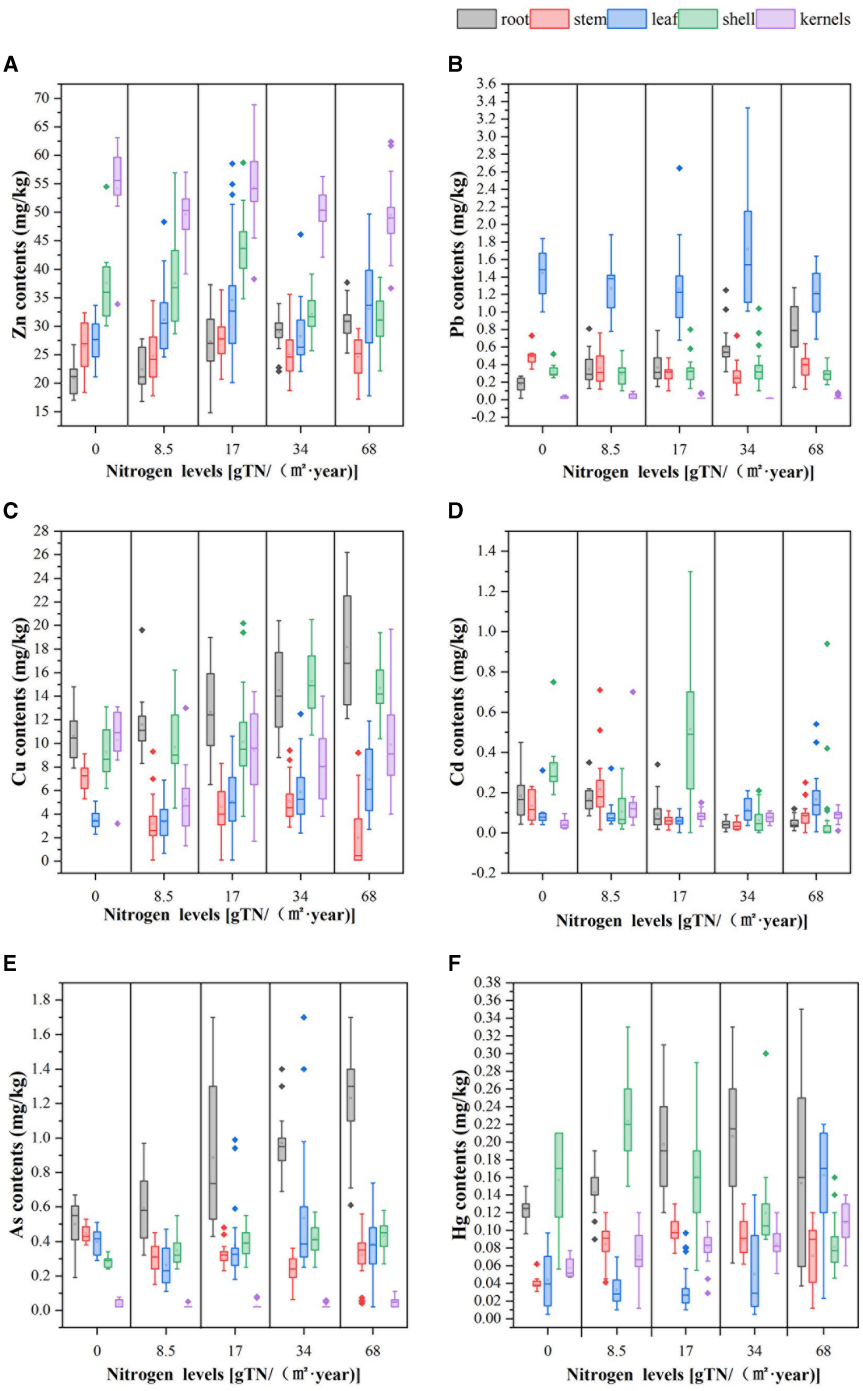
Distribution characteristics of heavy metal content in plant organs. **(A)** Zn contents, **(B)** Pb contents, **(C)** Cu contents, **(D)** Cd content, **(E)** As contents, and **(F)** Hg contents.

As can be seen from Figures 4A–F, the distribution of heavy metals in organs of peanut plants varied among treatments with different nitrogen application rates, which was mainly related to the different absorption capacities of heavy metals in different organs of peanut. In general, heavy metal content in peanut organs was higher at a high than low nitrogen application rate. Cu and Zn are essential trace elements for plant growth. However, when the concentration of Cu and Zn in the soil is higher than a certain critical value, they become toxic to plants. Excessive levels of Zn were found in a small number of peanut kernels in different biogas slurry treatments. The average Cu and Zn contents in peanut grain samples under different nitrogen application rates were significantly lower than 30 and 60 mg/kg, respectively, which are recommended by World Health Organization (69). According to the Limit of Contaminants in “Food Quality Safety and Food Contamination Limits” (54), the content of Pb in peanut grain samples under different nitrogen application rates was lower than the maximum permissible limit of 0.2 mg/kg. The toxicity of Cd would cause leaf yellowing, impaired plant growth, and a significant decrease in pod number (55). As can be seen from Figure 4D, the concentration of Cd in the peanut kernels was below the maximum permissible limit (0.5 mg/kg). Even though a non-essential element for crop growth, Cd can be absorbed by roots. Figure 4E shows that the content of As in peanut kernels under the nitrogen application rates was lower than the maximum permissible limit of 0.1 mg/kg. Cai et al. found that more As was accumulated in peanut shells than kernels (56), which is in line with the findings in the current study. Hg, a metal that is widely distributed in nature, is harmful to crop growth. As depicted in Figure 4F, the content of Hg in peanut kernel samples under different nitrogen application rates exceeded the standard of 0.02 mg/kg.

#### Enrichment and Transfer of Heavy Metals in the Soil-Peanut System

**Figure 5:**
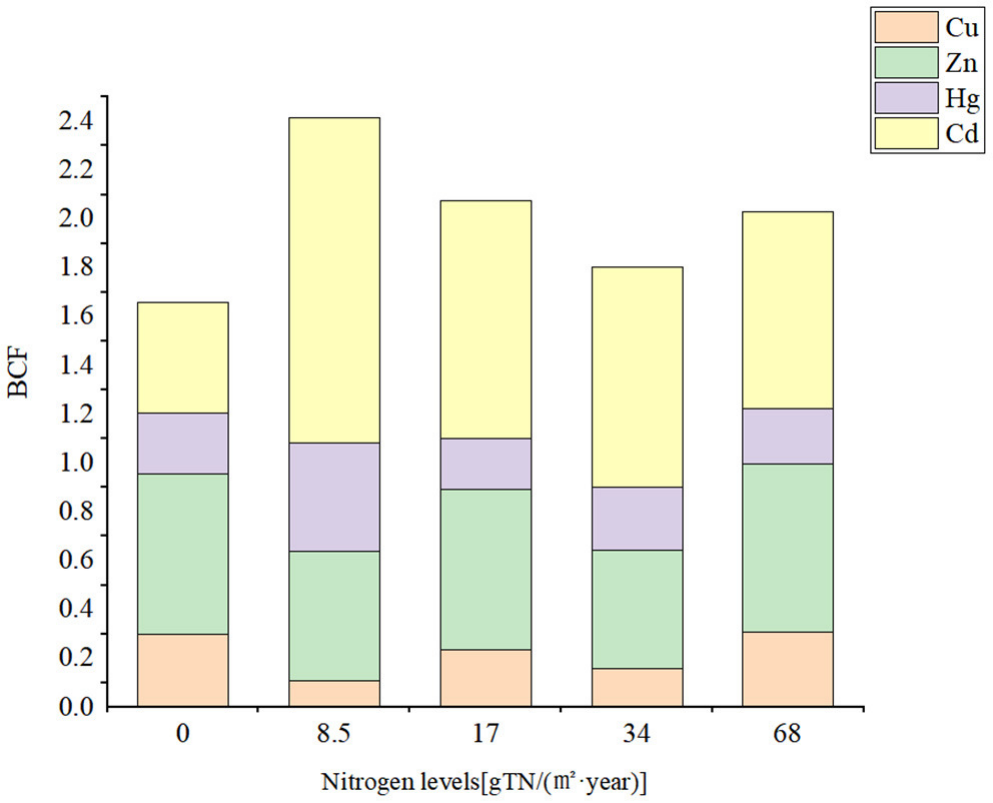
BCF (biological enrichment coefficient) of heavy metals in peanut kernels.

The accumulation characteristics of heavy metals in peanuts can be assessed by *BCF*, which reflects the ability of plants to accumulate heavy metals from the soil. *BCF* < 0.5 indicates low plant capacity to accumulate heavy metals; 0.5 < *BCF* <1 indicates some accumulation capacity of heavy metals, and *BCF*>1 shows that plants have a strong ability to accumulate heavy metals (57). As can be seen in Figure 5, the enrichment capacity of Cd and Zn in the peanut kernels is relatively high. The enrichment ability of Pb and As in the peanut plants was weak [*BCF*_(*Pb*)_ and *BCF*_(*As*)_ <0.01], and therefore the ecological risk of heavy metals was low. Zhao et al. (58) analyzed bio-accumulation of heavy metals in different parts of peanut, identifying strong bio-accumulation ability for Cu, Zn, and Cd, which was similar to the results of the present study.

**Figure 6:**
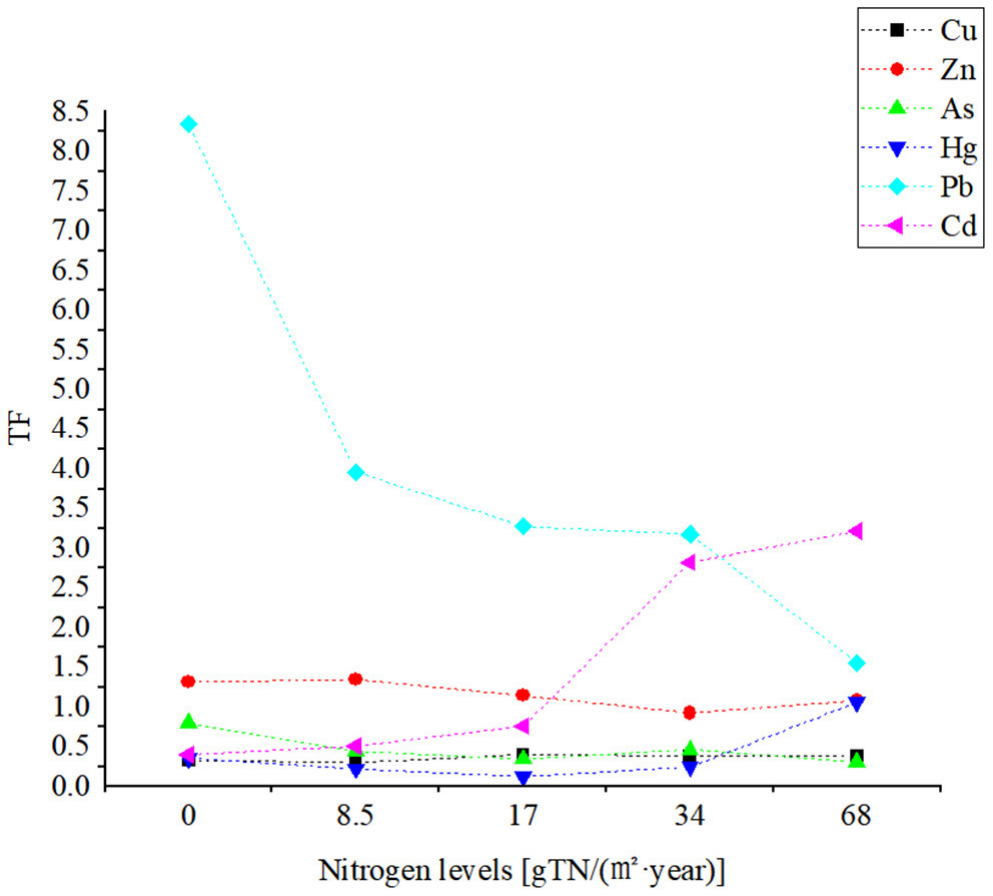
TF (transfer factor) of heavy metals in peanuts.

The ability of heavy metal translocations from roots to leaves can be expressed as *TF*. When *TF*>1, it can be inferred that plants can transfer the heavy metal elements from roots to the aboveground part. When *TF* <1, plants prevent the transport of heavy metals from roots to the aboveground parts (59). As seen in Figure 6, the TF of Pb in peanuts was high, in contrast, the TF of Cu and As was small, and hence the transfer ability was low. Under a high nitrogen application rate, the TF of Cd increased, suggesting a stimulating effect of high nitrogen supply on Cd distribution to leaves (60).

#### Correlation Analysis of Peanut and Soil Environment

**Figure 7:**
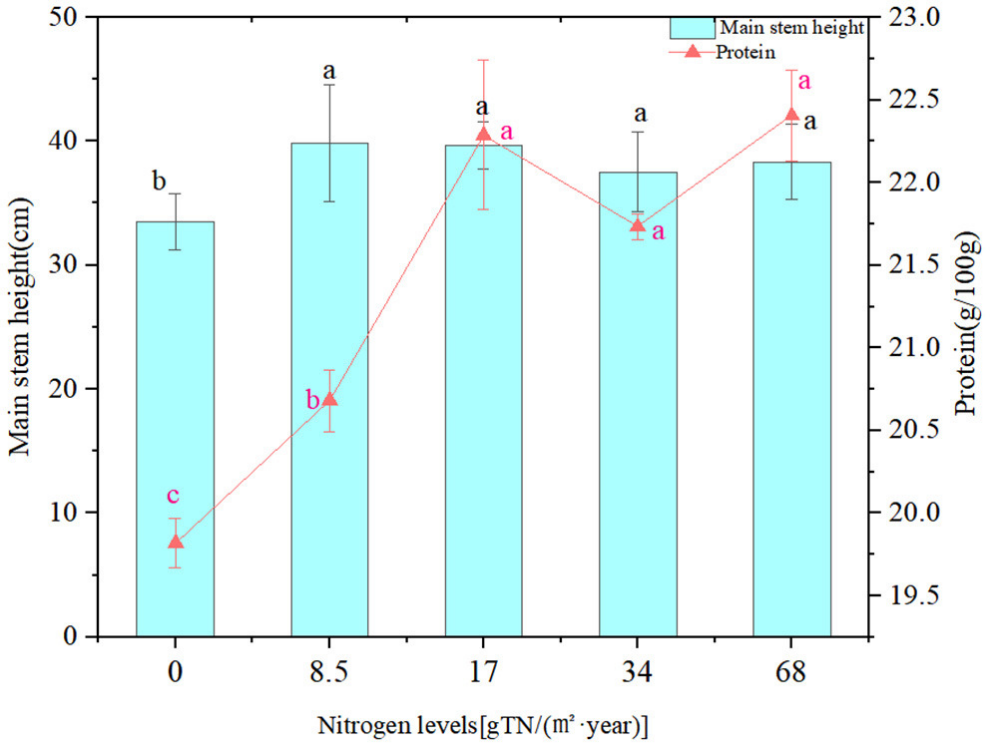
Effects of different fertilization treatments on main stem height and grain protein content of peanut. Lowercase letters a, b indicate significant differences at the level of 0.05. Marked with different letters means the difference is significant, marked with the same letter means the difference is not significant.

**Figure 8:**
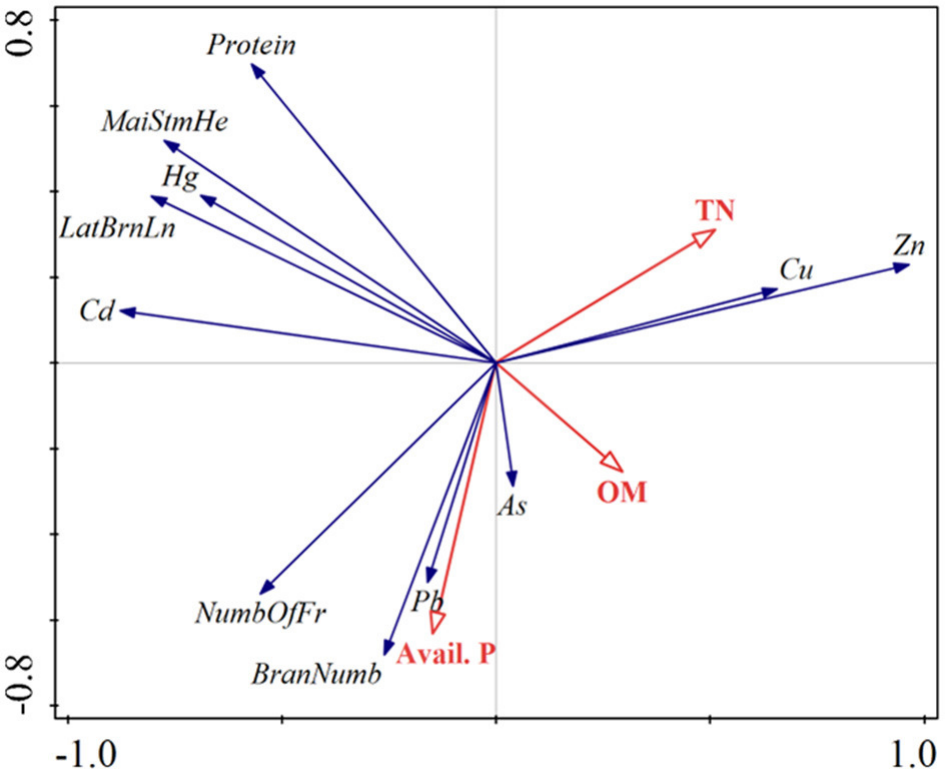
Redundancy analyses of the correlations between botanical character of peanut, protein and metal contents in peanut kernels (blue lines) and soil properties (red lines) using Canoco. MaiStmHe, main stem height; LatBrnLn, lateral branch length; BranNumb, branching number; NumbOfFr, number of fruit per plant.

Table 5 and Figure 7 show the peanut lateral branch length, branch number, fruit number per plant, main stem height, and protein under different fertilization treatments, respectively. It can be concluded that nitrogen application level has a significant influence on the protein content of peanut main stem height, lateral branch length (*p* < 0.05). With the increase of nitrogen application rate, the main stem height and protein content of peanut plants also showed an increasing trend. Through the redundancy analysis of the correlation between peanut and environmental factors, we revealed the relationship between soil properties and peanut traits after the application of biogas slurry (Figure 8). Total variation was found to be 140.79, and explanatory variables account for 82.6%. The decreasing order of effects of soil factors on the peanut kernels was TN > OM > Avail.P. The contents of Cu and Zn in peanut kernels were positively correlated with the soil TN after biogas slurry application. The branch number and fruit number per plant of peanut were positively correlated with the content of Avail.P in the soil after application of biogas slurry. Li et al. found that peanut protein content was negative correlated with soil OM and Avail.P. after biogas slurry application, which was consistent with the results of this study (61).

**Table 5:** Effects of different fertilization treatments on plant traits of peanut.

**Nitrogen levels g TN/(m^2^∙year)**	**Lateral branch length (cm)**	**Branching number**	**Number of fruit per plant**
0	31.50 ± 2.51^b^	6.86 ± 1.57^a^	12.86 ± 2.48^a^
8.5	37.17 ± 4.92^a^	7.33 ± 2.07^a^	13.67 ± 6.95^a^
17	36.17 ± 3.43^a^	5.67 ± 1.75^a^	11.00 ± 4.98^a^
34	35.17 ± 3.19^ab^	5.83 ± 1.17^a^	12.67 ± 6.41^a^
68	35.17 ± 2.99^ab^	7.17 ± 2.14^a^	15.83 ± 9.58^a^

*Lowercase letters a, b indicate significant differences at the level of 0.05. Marked with different letters means the difference is significant, marked with the same letter means the difference is not significant*.

### Health Risk Assessment of Biogas Slurry Used in Peanut Cultivation

Supplementary Table 2 presents the information on peanut consumers and their consumption and evaluates the intake of heavy metals by different groups via peanuts (62). The *EDI* (μg kg^−1^ d^−1^) values of peanuts from different fertilization treatments were shown in Table 6. Using the Provisional Tolerable Daily Intake (*PTDI*) as the reference standard, the *PTDI* values of Cu, Zn, Pb, Cd, As, and Hg in adults were 500, 1,000, 25, 0.83, 2.14, and 5 μg kg^−1^ d^−1^, respectively (63). If *EDI* value is less than *PTDI* value, it indicates that the health risk of heavy metals in peanuts is small. If *EDI* value is greater than *PTDI* value, it indicates that the health risk of heavy metals in peanuts is high. With different consumption levels, *EDI* values of all metals in children aged 2–6 years and standard adults were lower than *PTDI*. The *EDI* values of children aged 2–6 at the average consumption and high consumption level were significantly higher than those of standard adults. The *EDI* value of Cu and Zn ingested via peanuts in two groups was significantly higher than that of other metals. As can be seen from Figure 4, this was mainly because the contents of these two metals in peanut kernels were relatively high under different nitrogen application rates.

**Table 6:** Estimated daily intake of peanut under different fertilization treatments.

**Group**	**Consumption level**	**Metal**	**Nitrogen levels g TN/(m** ^**2**^ **∙** **year)**				
			**0**	**8.5**	**17**	**34**	**68**
2-to-6-year-old children	Mean	EDI_(Cu)_	1.2326	0.5292	1.0264	0.8753	1.0811
		EDI_(Zn)_	6.2426	5.4274	6.0275	5.5135	5.3584
		EDI_(Pb)_	0.0030	0.0047	0.0022	0.0022	0.0022
		EDI_(Cd)_	0.0049	0.0121	0.0089	0.0081	0.0097
		EDI_(As)_	0.0041	0.0022	0.0022	0.0022	0.0053
		EDI_(Hg)_	0.0063	0.0078	0.0093	0.0092	0.0119
	High	EDI_(Cu)_	18.4887	7.9373	15.3966	13.1300	16.2165
		EDI_(Zn)_	93.6386	81.4115	90.4127	82.7018	80.3755
		EDI_(Pb)_	0.0449	0.0701	0.0328	0.0328	0.0328
		EDI_(Cd)_	0.0742	0.1819	0.1339	0.1216	0.1458
		EDI_(As)_	0.0617	0.0328	0.0328	0.0328	0.0801
		EDI_(Hg)_	0.0941	0.1165	0.1396	0.1384	0.1783
Standard adult	Mean	EDI_(Cu)_	0.5440	0.2336	0.4530	0.3863	0.4772
		EDI_(Zn)_	2.7553	2.3955	2.6604	2.4335	2.3650
		EDI_(Pb)_	0.0013	0.0021	0.0010	0.0010	0.0010
		EDI_(Cd)_	0.0022	0.0054	0.0039	0.0036	0.0043
		EDI_(As)_	0.0018	0.0010	0.0010	0.0010	0.0024
		EDI_(Hg)_	0.0028	0.0034	0.0041	0.0041	0.0052
	High	EDI_(Cu)_	6.4310	2.7609	5.3555	4.5671	5.6407
		EDI_(Zn)_	32.5709	28.3178	31.4488	28.7666	27.9575
		EDI_(Pb)_	0.0156	0.0244	0.0114	0.0114	0.0114
		EDI_(Cd)_	0.0258	0.0633	0.0466	0.0423	0.0507
		EDI_(As)_	0.0215	0.0114	0.0114	0.0114	0.0279
		EDI_(Hg)_	0.0327	0.0405	0.0486	0.0481	0.0620

*The content of heavy metals in peanut kernels was calculated by the average value after discarding the outliers, and the undetected data was calculated by half of the detection limit. The detection limit of Cu, Zn, Pb, Hg, As and Cd was 0.2, 1, 0.04, 0.01, 0.04 and 0.003 mg/kg.

For the same group, the *EDI* value of different metals was greater at high consumption than average consumption level. Although *EDI*_(*Hg*)_ was less than *PTDI*, Hg in peanut kernels under different nitrogen application rates exceeded the standard according to the results shown in section Distribution of Heavy Metals in Plant Organs. This phenomenon is noteworthy because children at high consumption levels are more likely to ingest Hg by eating peanuts. Hg can cause teratogenicity and chromosomal abnormalities. Chronic mercury poisoning damages the human nervous system. Methylmercury is easily transferred from mother to fetus through the placenta (64). Chen et al. (65) assessed the dietary exposure risk of Cd in peanuts in China and found that the risk from overall dietary exposure is low. At the general consumption level, the exposure of the population is far lower than the provisional tolerable monthly intake (*PTMI*) value; however, at a high consumption level, 5% of individuals in the population are close to or exceed *PTMI* value, which carries potential risks.

As can be seen from Table 7, under different fertilization treatments, the *THQ* value of peanut intake by different groups with different consumption levels was <1. Similar to the *EDI* value, the *THQ* value for children aged 2–6 was higher than that of standard adults at both average and high consumption levels. For the same group, *THQ* values of different metals were larger at high than average consumption level. The *THQ* value of Cu, As, Hg, and Zn ingested via peanuts in two groups was higher than that of other metals. Although Cu and Zn are essential trace elements for humans, excessive Cu and Zn can adversely affect human health. Excess Cu causes liver and gallbladder problems. Excessive intake of Zn may cause deficiency of other nutrients, such as Ca, P, and Fe and lead to poisoning. Arsenic may cause a systemic disease associated with skin damage, which can harm human skin, respiratory, digestive, urinary, cardiovascular, nervous, hematopoietic, and other systems. The *TTHQ* was higher under high than low nitrogen application. The *TTHQ* values for children aged 2–6 with high consumption levels were >1 (except at 8.5 g TN/m^2^·year), and the *TTHQ* values of other consumer groups were all <1. *TTHQ* above 1 indicates that this population is likely to have significant adverse effects due to heavy metal ingestion, and this possibility increases with an increase in *TTHQ* value at the high nitrogen application rate. Similarly, some studies have also shown potential pollution by the use of biogas slurry in agriculture. Zhang et al. (66) studied the effects of biogas slurry on vegetable crops cultivated in open fields. The results showed that applying digested pig slurry in the vegetable fields could improve soil fertility. The contents of Hg, Zn, Cr, Cd, Pb, and Cu in fertilized soil did not exceed the maximum allowable content for vegetable crop soils in China. However, due to the repeated use of digested pig slurry, the high zinc accumulation should be of concern. Shamsollahi et al. (16) used raw sludge and anaerobically digested sludge for lettuce cultivation. It was found that the application of anaerobically digested sludge could increase the bioaccumulation rate of heavy metals in crops and increase risks to human health. Bian et al. (67) studied the health risks of multi-faceted exposure to heavy metals (Pb, Cd, Cr, Zn, Cu, and As) via water, soil, air, and locally produced food in the Taihu Lake Basin. Their results showed that Zn, Pb, and Cd in soils irrigated with biogas slurry exceeded the soil quality standard values, and the vegetables and grains were contaminated by Pb and Cd, exceeding the allowable limits. Xie et al. (68) applied biogas slurry repeatedly for 4 years (N1) and 10 years (N2) in two vegetable plots to measure heavy metal content (Zn, Cu, Cr, and Pb) and assess the ecological and health risks of short- and long-term fertilization. The results showed long-term fertilization increased the content of heavy metals. In general, N2 had a higher non-carcinogenic and carcinogenic risk index compared with N1. Consistent with the results of the present study, Xie et al. also concluded that children are more sensitive to environmental changes than adults, and more attention should be paid to protecting their health.

**Table 7:** Total Hazard Quotient of peanut under different fertilizer treatments.

**Group**	**Consumption level**	**Metal**	**Nitrogen levels g TN/(m** ^**2**^ **∙** **year)**				
			**0**	**8.5**	**17**	**34**	**68**
2-to-6-year-old children	Mean	THQ_(Cu)_	0.0308	0.0132	0.0257	0.0219	0.0270
		THQ_(Zn)_	0.0208	0.0181	0.0201	0.0184	0.0179
		THQ_(Pb)_	0.0009	0.0013	0.0006	0.0006	0.0006
		THQ_(Cd)_	0.0016	0.0040	0.0030	0.0027	0.0032
		THQ_(As)_	0.0137	0.0073	0.0073	0.0073	0.0178
		THQ_(Hg)_	0.0125	0.0155	0.0186	0.0185	0.0238
		TTHQ	0.0804	0.0595	0.0753	0.0693	0.0903
	High	THQ_(Cu)_	0.4622	0.1984	0.3849	0.3282	0.4054
		THQ_(Zn)_	0.3121	0.2714	0.3014	0.2757	0.2679
		THQ_(Pb)_	0.0128	0.0200	0.0094	0.0094	0.0094
		THQ_(Cd)_	0.0247	0.0606	0.0446	0.0405	0.0486
		THQ_(As)_	0.2057	0.1094	0.1094	0.1094	0.2670
		THQ_(Hg)_	0.1882	0.2331	0.2792	0.2768	0.3567
		TTHQ	1.2059	0.8929	1.1288	1.0400	1.3549
Standard adult	Mean	THQ_(Cu)_	0.0136	0.0058	0.0113	0.0097	0.0119
		THQ_(Zn)_	0.0092	0.0080	0.0089	0.0081	0.0079
		THQ_(Pb)_	0.0004	0.0006	0.0003	0.0003	0.0003
		THQ_(Cd)_	0.0007	0.0018	0.0013	0.0012	0.0014
		THQ_(As)_	0.0061	0.0032	0.0032	0.0032	0.0079
		THQ_(Hg)_	0.0055	0.0069	0.0082	0.0081	0.0105
		TTHQ	0.0355	0.0263	0.0332	0.0306	0.0399
	High	THQ_(Cu)_	0.1608	0.0690	0.1339	0.1142	0.1410
		THQ_(Zn)_	0.1086	0.0944	0.1048	0.0959	0.0932
		THQ_(Pb)_	0.0045	0.0070	0.0033	0.0033	0.0033
		THQ_(Cd)_	0.0086	0.0211	0.0155	0.0141	0.0169
		THQ_(As)_	0.0716	0.0380	0.0380	0.0380	0.0929
		THQ_(Hg)_	0.0655	0.0811	0.0971	0.0963	0.1241
		TTHQ	0.4194	0.3106	0.3926	0.3617	0.4713

## Conclusions

In this study, three indicators, i.e., soil ecological risk, peanut growth risk, and human health risk, were comprehensively evaluated to assess the safety of biogas slurry application as a nutrient supplier. According to the results achieved herein, the application of biogas slurry affected the physical and chemical properties of soil, plants' traits and protein content of peanuts, and the content of heavy metals in different parts of soil and in peanuts. As a nutrient source for peanut cultivation, biogas slurry promotes the growth of peanuts; however, it also strengthens the accumulation of heavy metals in the plants and exacerbates soil quality. Moreover, the use of peanuts, fertilized with biogas slurry, can transfer such heavy metals to the bodies of human beings. This risk should not be overlooked because it becomes more pronounced with increased biogas slurry application and will be more dangerous for children. This poses a serious challenge to the replacement of synthetic fertilizers with organic fertilizers in the future. Therefore, long-term monitoring is needed, one the one hand, to understand food safety under crop cultivations with organic fertilizers, such as biogas slurry, and on the other hand, to find solutions over how to reduce and control heavy metals in biogas slurry when it is intended to be applied on farmland.

## Data Availability Statement

The original contributions presented in the study are included in the article/Supplementary Material, further inquiries can be directed to the corresponding author/s.

## Author Contributions

QL: methodology and investigation. ZZ: writing—original draft, conceptualization, methodology, and data curation. ZX: validation and formal analysis. DL: investigation. ZW: data analysis. YR: supervision. ZM: supervision and project administration. LH: writing—review and editing, supervision, project administration, and funding acquisition. All authors contributed to the article and approved the submitted version.

## Funding

This work was financially supported by the National Natural Science Foundation of China (Grants No. 31902208), the fundamental research funds for the Central Non-profit Scientific Institution (161001202003_02201), the Sichuan Science and Technology Program (2018JY0543), and the Agricultural Science and Technology Innovation Project of Chinese Academy of Agricultural Sciences (CAAS-ASTIP-2016-BIOMA).

## Conflict of Interest

The authors declare that the research was conducted in the absence of any commercial or financial relationships that could be construed as a potential conflict of interest.

## Publisher's Note

All claims expressed in this article are solely those of the authors and do not necessarily represent those of their affiliated organizations, or those of the publisher, the editors and the reviewers. Any product that may be evaluated in this article, or claim that may be made by its manufacturer, is not guaranteed or endorsed by the publisher.

## Abbreviations

Cu: copper
EU: European Union
Zn: zinc
WHO: World Health Organization
Pb: lead
USEPA: U.S. Environmental Protection Agency
Cr: chromium
*IPI*_*N*_: integrated pollution index
Cd: cadmium
*RI*: potential ecological risk index
Hg: mercury
*BCF*: biological enrichment factor
As: arsenic
*TF*: transfer factor
TN: total nitrogen
*EDI*: estimated daily intake
TP: total phosphorus
*THQ*: total hazard quotient
TK: total potassium
*TTHQ*: total target hazard quotient
OM: organic matter
*PTDI*: provisional tolerable daily intake
Avail. N.: alkali-hydrolyzed nitrogen
*PTMI*: provisional tolerable monthly intake
Avail.P.: available phosphorus
Avail.K.: available potassium.
