# Supplementary material for: Comprehensive Risk Assessment of Applying Biogas Slurry in Peanut Cultivation
Source: Front Nutr. 2021 Oct 14;8:702096. doi: 10.3389/fnut.2021.702096 (PMC8552993; doi:10.3389/fnut.2021.702096)
Supplement: Supplementary file 1 [file Data_Sheet_1.pdf]

Supplementary Material

1 Supplementary Data Resource

References from which species lists of native and invasive seed plants of the 67 nature reserves were compiled.

[1] Beijing Beihuashan nature reserve scientific survey group (2003) Beijing Baihuashan nature reserve scientific survey report.

[2] Che X (2006) Species diversity and structure of forest communities in Tongguling Nature Reserve. Haikou: Hainan University Thesis for Master Degree.

[3] Chen S (2006) Scientific survey of the Xiaerxili Nature Reserve, Xinjiang. Urumqi: Xinjiang Science and Technology Press.

[4] Cheng J (2013) Scientific survey of the national Liupanshan Nature Reserve. Beijing: Science Press.

[5] Editorial board of this book (2009) Resources investigation report of Fangcheng golden camellia National Nature Reserve, Guangxi.

[6] Gao H, Xu W, Lin C, Liu Y (2008) Flora of the seed plants in Huaping National Nature Reserve, Guangxi. Guihaia, 28: 780-784.

[7] Gao Y, Sun X (1991) The list of flowering plants in Lianhua Mountain. Journal of Gansu Agricultural University, 26: 207-215.

[8] Guangdong Forestry Department (1993) Memoir of scientific research in Chebaling Nature Reserve. Guangzhou: Guangdong Science and Technology Press.

[9] Guangxi comprehensive investigation group for Huaping (1986) Scientific report on Huaping, Guangxi. Jinan: Shandong Science and Technology Press.

[10] Guizhou Forestry Department (2000) Scientific survey of the Fodingshan Nature Reserve in Guizhou. Beijing: China Forestry Press.

[11] Huang J, Li X, Qian J (1996) Flora of Saihanba. Beijing: China Science and Technology Press.

[12] Huang K (2004) Research on seed plant diversity in Diaoluoshan Nature Reserve, Hainan Island. Guangzhou: Sun Yat-Sen University Thesis for Master Degree.

[13] Huang X (2010) Species list of vascular plants in Dali Cangshanerhai National Nature Reserve. Kunming: Yunnan University Press.

[14] Hunan Forestry Bureau (1987) Nature resources in Hunan Bamianshan nature reserve. Published by Hunan Forestry Bureau.

[15] Jiang Z (2005) Biodiversity of the Qingmuchuan Nature Reserve, Saanxi, China. Beijing: Tsinghua University Press.

[16] Jin X, Ding B, Zheng C, Ye Z, Chen X (2004) The floristic analysis of seed plants in Baishanzu Nature Reserve from Zhejiang Province. Acta Botanica Yunnanica, 26: 605-618.

[17] Juan Wang, Ma Q, Du F (2006) Flora diversity characteristics of plants of Dawei Mountain National Nature Reserve in Yunnan Province, China. Scienta Silvae Sinicar, 42: 7-15.

[18] Li Z (1994) Species list of Longqi Mountain. Beijing: China Science and Technology Press.

[19] Lin P (2001) Report of scientific research in Liangyeshan Nature Reserve. Xiamen: Xiamen University Press.

[20] Lin P (2002) Report of scientific research in Tianbaoyan Nature Reserve. Xiamen: Xiamen University Press.

[21] Lin P (2003) Report of scientific research in Daiyunshan Nature Reserve. Xiamen: Xiamen University Press.

[22] Lin P (2003) Report of scientific research in Mangdangshan Nature Reserve. Xiamen: Xiamen University Press.

[23] Lin P, Li Z, Zhang J (2005) Report of scientific research in Junzifeng Nature Reserve. Xiamen: Xiamen University Press.

[24] Liu F, Tao G, Wang D (2008) Investigation on the alien invasive plants in Nabanhe Nature Reserve and precautionary measures. Forest Inventory and Planning, 33: 112-117.

[25] Liu K, Hou B (1991) A study on forest flora of Taoyuandong Nature Reserve, Hunan. Journal of Wuhan Botanical Research, 9: 53-61.

[26] Liu L (2006) Studies on plant diversity of Fenghuangshan in Liaoning Province. Changchun: Northeast Normal University.

[27] Liu N, Ma C (1997) Gahai-Zecha Nature Reserve. Beijing: China Forestry Press.

[28] Lou L, Jin S (2000) Scientific survey of the Gutianshan National Nature Reserve. Hangzhou: Zhejiang Science and Technology Press.

[29] Luo Y (2012) Scientific survey of the Xishui Nature Reserve. Guiyang: Guizhou Science and Technology Press.

[30] Lu P (2010) Study on plant diversity and conservation in Hanma National Nature Reserve. Harbin: Northeast Forestry University.

[31] Ma J (1993) Researches in Liangshui Nature Reserve. Haerbin: Northeast Forestry University Press.

[32] Ma S (1998) Jinfoshan nature reserve scientific survey report.

[33] Mangshan Forestry Bureau. (1993) Scientific survey of the Mangshan Nature Reserve. Published by Mangshan Forestry Bureau.

[34] Meng F (2006) Study on species diversity and conservation the flora of Yuanbaoshan Nature Reserve, Guangxi, China. Guilin: Guangxi Normal University.

[35] Nabanhe National Nature Reserve Management Station. (2006) Nabanhe National Nature Reserve in Watershed of Nabanhe, Xishuangbanna. Kunming: Yunnan Science and Technology Press.

[36] Peng M, Wang C, Dang C (2006) Biodiversity and Conservation in Yaoshan Nature Reserve, Yunnan. Beijing: Science Press.

[37] Qi C, Yu X, Cao T, Zhou J (1994) Flora of Hunan Badagongshan Mountains and its phytogeograpgical significance. 16: 321-332.

[38] Qin W, Wang Z, Xu W, Jiang M (2008) Investigation and analysis on alien invasiveplants in three national nature reserves in Hainan Province. Journal of Plant Resources and Environment, 17: 44-49. [Tongguling]

[39] Qinghai Forestry Bureau. (1990) Qinghai Mengda Nature Reserve. Xining: Qinghai People's Publishing House.

[40] Science and technology commission of Fujian Province (1993) Report of scientific research in Wuyishan Nature Reserve. Fuzhou: Fujian Science and Technology Press.

[41] Shaanxi Forestry Bureau (1987) Scientific survey of the Taibaishan Nature Reserve. Shaanxi Normal University Press.

[42] Shangguan T, Zhang F, Qiu F, Zhang F (1999) Studies on flora diversity of the seed plants in Luya Mountain Nature Reserve, Shanxi. Journal of Wuhan Botanical Research, 17: 323-331.

[43] Song C (1994) Scientific survey of the Funiushan Nature Reserve. Beijing: China Forestry Press.

[44] Song C (1994) Scientific survey of the Jigongshan Nature Reserve. Beijing: China Forestry Press.

[45] Song C (1997) Scientific survey of the Qingliangfeng Nature Reserve, Zhejiang. Beijing: China Forestry Press.

[46] Song C, Liu S (1999) Scientific survey of the Houhe Nature Reserve. Beijing: China Forestry Press.

[47] Song C, Qu W (1996) Scientific survey of the Dongzhai Bird Nature Reserve. Beijing: China Forestry Press.

[48] Song C, Qu W (1996) Scientific survey of the Taihangshan Macaque Nature Reserve. Beijing: China Forestry Press.

[49] Sun J, Yang G, Chen Y, Teng Y, Yan D, Yi S (2009) Exotic plants in the Jinfo Mountain Nature Reserve: Species ascertain and their distribution. Acta Pratacul Turae Sinica, 18: 34-42.

[50] Taizi Mountain Nature Reserve (2007) Report of scientific research in Taizi Mountain Nature Reserve, Gansu.

[51] Tan W (2005) Conservation and Research of biodiversity in Cenwanglao Mountain Nature Reserve, Guangxi. Beijing: China Environmental Science Press.

[52] Wang J, Du F, Yang Y, Tian K, Wang Y (2010) Scientific survey of the Upper Mekong River Nature Reserve. Beijing: Science Press.

[53] Wang T (2007) Studies on plant diversity and conservation in Tianjin Baxianshan Nature Reserve. Beijing: Beijing Forestry University.

[54] Wang X (2011) Scientific survey of the national Helanshan Nature Reserve, Ningxia. Yinchuan: Sun Press.

[55] Wu Y, Zhao J, Cheng J (2006) Scientific survey and biodiversity research in Maojingba Nature Reserve, Hebei. Beijing: Science Press.

[56] Yang Q, Liu J, Wang Y (2007) Report of scientific research in Qilian Mountain National Nature Reserve, Gansu. Lanzhou: Gansu Science and Technology Press.

[57] Yaoluoping nature reserve management committee (2001) The overall planning of Yaoluoping nature reserve.

[58] Ye Y, Qu W, Huang Y (2002) Scientific survey of the Liankangshan Nature Reserve. Science Press.

[59] Yin X (2007) Studies on the Flora of Vascular Plant and Wild Plant Resources of South Dahong Mountain. Chinese Academy of Science.

[60] Yuan Y, Song C (1998) Scientific survey of the Baishilazi Nature Reserve. Beijing: China Forestry Press.

[61] Zeng Q (1995) A list of bio-species in Jianfengling of China. Beijing: China Forestry Press.

[62] Zhang L, Guo C (1996) Scientific survey of the Laotudingzi Nature Reserve.

[63] Zhang X (2012) Flora of Taishan. Jinan: Shandong Science and Technology Press.

[64] Zhao D (2008) Botanical illustration of Changbaishan. Shenyang: Shenyang Press.

[65] Zhao J, Wu Y, Li P (2005) Scientific survey and biodiversity research in Mulan Weichang Nature Reserve, Hebei. Beijing: Science Press.

[66] Zhao X (2007) Study on the flora of seed plants in the Wula Mountain. Huhhot: Inner Mongolia Agricultural University.

[67] Zheng Y (1999) Introduction to Mulun Karst Forest. Beijing: Science Press.

[69] Zheng Y, Qiu F (1998) Scientific survey of the Yading Nature Reserve.

[70] Zhu D, Zhao Z, Du X, Sang T (2010) A preliminary floristic study of seed plants in Longxi-Hongkou National Nature Reserve. Journal of Sichuan Forestry Science and Technology, 31: 43-47.

[71] Zhu Z, Song C (1999) Scientific survey of the Shennongjia Nature Reserve. Beijing: China Forestry Press.

2 Supplementary Tables

Table S1 The coordinates, the number and the proportion of invasive plants in each of the 67 nature reserves.

Nature reserve	Longitude	Latitude	Number of invasive plant species	Proportion of invasive plant species (%)
Xiaerxili	43.2	83.0	19	3.16
Qilian Mountain	38.1	98.1	18	1.58
Upper Mekong	24.0	99.7	4	0.38
Cangshanerhai	25.8	100.1	42	2.47
Yading	29.0	100.2	17	1.98
Nabanhe	22.2	100.6	38	3.39
Gaihai-Zecha	34.3	102.5	2	0.46
Mengda	35.8	102.7	5	1.10
Yaoshan	27.3	103.1	36	2.65
Taizi Mountain	35.3	103.2	10	1.31
Longxi-Hongkou	31.2	103.6	30	1.59
Lianhua Mountain	34.9	103.8	13	1.95
Dawei Mountain	22.9	103.9	56	1.50
Qingmuchuan	32.9	105.6	38	2.83
Cenwanglao Mountain	24.3	106.2	52	2.73
Xishui	28.3	106.2	9	1.06

Liupanshan	35.5	106.3	8	1.40
Helanshan	39.0	106.5	8	1.36
Jinfoshan	29.2	107.2	129	3.55
Mulun	25.2	107.8	17	2.20
Taibaishan	34.0	107.8	5	0.64
Fangcheng	21.8	108.1	35	2.71
Fodingshan	27.3	108.1	16	1.56
Jianfengling	18.6	108.9	80	3.58
Wula Mountain	40.7	109.1	7	1.51
Yuanbaoshan	25.5	109.2	21	1.62
Huaping	25.6	109.9	17	1.35
Diaoluoshan	18.7	109.9	29	1.63
Badagongshan	29.7	109.9	32	2.54
Shennongjia	31.5	110.3	34	1.59
Houhe	30.1	110.4	34	2.07
Tongguling	19.7	111.0	60	6.81
Luya Mountain	38.7	112.0	3	2.13
Funiushan	33.4	112.2	36	1.54
Manghe	35.3	112.4	31	4.35
Taihangshan	35.1	112.9	46	3.04

Mangshan	25.0	112.9	36	1.98
Dahong Mountain	31.3	113.0	37	3.83
Bamianshan	26.0	113.7	20	1.71
Taoyuandong	26.4	114.0	33	2.51
Jigongshan	31.8	114.1	12	0.83
Chebaling	24.5	114.2	26	2.17
Dongzhai	31.8	114.4	43	2.65
Liankangshan	31.6	114.8	37	2.35
Baihuashan	40.0	115.6	22	3.01
Yaoluoping	31.0	116.1	34	4.15
Liangyeshan	25.2	116.1	60	4.29
Weichang	42.0	116.9	12	1.73
Taishan	36.4	117.0	44	4.70
Junzifeng	26.5	117.2	39	2.81
Longqi Mountain	26.6	117.3	46	3.65
Saihanba	42.4	117.4	11	1.96
Baxianshan	40.2	117.4	6	1.90
Tianbaoyan	25.9	117.5	31	3.12
Wuyishan	27.7	117.7	61	3.09
Maojingba	41.5	118.0	15	1.83

Mangdangshan	26.7	118.1	41	3.52
Daiyunshan	25.7	118.2	49	3.45
Gutianshan	29.3	118.2	39	2.83
Qingliangfeng	30.2	119.0	41	2.98
Baishanzu	27.8	119.5	60	3.48
Hanma	51.4	122.6	1	0.42
Fenghuangshan	40.4	124.1	31	3.56
Baishilazi	40.9	124.8	21	2.47
Laotudingzi	41.3	124.9	26	3.07
Changbaishan	42.7	125.9	23	1.86
Liangshui	47.2	128.9	4	1.24
