# Supplementary material for: Comprehensive Risk Assessment of Applying Biogas Slurry in Peanut Cultivation
Source: Front Nutr. 2021 Oct 14;8:702096. doi: 10.3389/fnut.2021.702096 (PMC8552993; doi:10.3389/fnut.2021.702096)
Supplement: Supplementary file 2 [file Data_Sheet_2.docx]

Table S1

Screening value of soil pollution risk in agricultural land(excerpt)

| Contaminant project | | Risk screening value | | | |
| --- | --- | --- | --- | --- | --- |
|  |  | pH≤5.5 | 5.5＜pH≤6.5 | 6.5＜pH≤7.5 | pH＞7.5 |
| Cd  (mg/kg) | Paddy field | 0.3 | 0.4 | 0.6 | 0.8 |
|  | Other | 0.3 | 0.3 | 0.3 | 0.6 |
| Hg  (mg/kg) | Paddy field | 0.5 | 0.5 | 0.6 | 1.0 |
|  | Other | 1.3 | 1.8 | 2.4 | 3.4 |
| As  (mg/kg) | Paddy field | 30 | 30 | 25 | 20 |
|  | Other | 40 | 40 | 30 | 25 |
| Pb  (mg/kg) | Paddy field | 80 | 100 | 140 | 240 |
|  | Other | 70 | 90 | 120 | 170 |
| Cr  (mg/kg) | Paddy field | 250 | 250 | 300 | 350 |
|  | Other | 150 | 150 | 200 | 250 |
| Cu  (mg/kg) | Paddy field | 150 | 150 | 200 | 200 |
|  | Other | 50 | 50 | 100 | 100 |
| Zn  (mg/kg) |  | 200 | 200 | 250 | 300 |

Table S2

Peanut consumption groups and consumption amount

| Group | Weight/kg | Amount of Peanut Consumption/g·d^-1^ | |
| --- | --- | --- | --- |
|  |  | Mean-Level Consumption | High-Level Consumption |
| 2-to-6-year-old children | 15.18 | 1.66 | 24.90 |
| Standard adult* | 62.57 | 3.02 | 35.70 |

*Adult male who engages in light physical labor.

Table S3

Classification and expression of environmental quality of heavy metals in soil

| Indicator | First-class | Second-class | Third-class | Fourth-class | Fifth-class |
| --- | --- | --- | --- | --- | --- |
| $PI_{i}$  Description | $PI_{i}\leq1$  Clean | $1<PI_{i}\leq$2  Minor pollution | $2<PI_{i}\leq$3 Mild contamination | $3<PI_{i}\leq$5  [Middle](../AppData/Roaming/Microsoft/AppData/Local/youdao/dict/Application/8.9.5.0/resultui/html/index.html#/javascript:;) [level](../AppData/Roaming/Microsoft/AppData/Local/youdao/dict/Application/8.9.5.0/resultui/html/index.html#/javascript:;) [pollution](../AppData/Roaming/Microsoft/AppData/Local/youdao/dict/Application/8.9.5.0/resultui/html/index.html#/javascript:;) | $PI_{i}>5$  [Heavy](../AppData/Roaming/Microsoft/AppData/Local/youdao/dict/Application/8.9.5.0/resultui/html/index.html#/javascript:;) [pollution](../AppData/Roaming/Microsoft/AppData/Local/youdao/dict/Application/8.9.5.0/resultui/html/index.html#/javascript:;) |
| $\mathrm{IP}I_{N}$  Description | $\mathrm{IP}I_{N}\leq0.7$  Clean | $0.7<IPI_{N}\leq1$  Mildly clean | $1<IPI_{N}\leq$2  Mild contamination | $2<IPI_{N}\leq3$  [Middle](../AppData/Roaming/Microsoft/AppData/Local/youdao/dict/Application/8.9.5.0/resultui/html/index.html#/javascript:;) [level](../AppData/Roaming/Microsoft/AppData/Local/youdao/dict/Application/8.9.5.0/resultui/html/index.html#/javascript:;) [pollution](../AppData/Roaming/Microsoft/AppData/Local/youdao/dict/Application/8.9.5.0/resultui/html/index.html#/javascript:;) | $\mathrm{IP}I_{N}>3$  [Heavy](../AppData/Roaming/Microsoft/AppData/Local/youdao/dict/Application/8.9.5.0/resultui/html/index.html#/javascript:;) [pollution](../AppData/Roaming/Microsoft/AppData/Local/youdao/dict/Application/8.9.5.0/resultui/html/index.html#/javascript:;) |
| $\mathrm{RI}$  Description | $RI<150$  Low | $150\leq RI<300$  Middle | $300\leq RI<600$  Higher | $RI\geq600$  [The](../AppData/Roaming/Microsoft/AppData/Local/youdao/dict/Application/8.9.5.0/resultui/html/index.html#/javascript:;) [highest](../AppData/Roaming/Microsoft/AppData/Local/youdao/dict/Application/8.9.5.0/resultui/html/index.html#/javascript:;) |  |
